# Machine learning models trained on synthetic datasets of multiple sample sizes for the use of predicting blood pressure from clinical data in a national dataset

**DOI:** 10.1371/journal.pone.0283094

**Published:** 2023-03-16

**Authors:** Anmol Arora, Ananya Arora

**Affiliations:** School of Clinical Medicine, University of Cambridge, Cambridge, United Kingdom; Jeonbuk National University, REPUBLIC OF KOREA

## Abstract

**Introduction:**

The potential for synthetic data to act as a replacement for real data in research has attracted attention in recent months due to the prospect of increasing access to data and overcoming data privacy concerns when sharing data. The field of generative artificial intelligence and synthetic data is still early in its development, with a research gap evidencing that synthetic data can adequately be used to train algorithms that can be used on real data. This study compares the performance of a series machine learning models trained on real data and synthetic data, based on the National Diet and Nutrition Survey (NDNS).

**Methods:**

Features identified to be potentially of relevance by directed acyclic graphs were isolated from the NDNS dataset and used to construct synthetic datasets and impute missing data. Recursive feature elimination identified only four variables needed to predict mean arterial blood pressure: age, sex, weight and height. Bayesian generalised linear regression, random forest and neural network models were constructed based on these four variables to predict blood pressure. Models were trained on the real data training set (n = 2408), a synthetic data training set (n = 2408) and larger synthetic data training set (n = 4816) and a combination of the real and synthetic data training set (n = 4816). The same test set (n = 424) was used for each model.

**Results:**

Synthetic datasets demonstrated a high degree of fidelity with the real dataset. There was no significant difference between the performance of models trained on real, synthetic or combined datasets. Mean average error across all models and all training data ranged from 8.12 To 8.33. This indicates that synthetic data was capable of training equally accurate machine learning models as real data.

**Discussion:**

Further research is needed on a variety of datasets to confirm the utility of synthetic data to replace the use of potentially identifiable patient data. There is also further urgent research needed into evidencing that synthetic data can truly protect patient privacy against adversarial attempts to re-identify real individuals from the synthetic dataset.

## Introduction

Broadly, there are two types of machine learning: generative and deductive. Deductive machine learning models are those which analyse datasets to yield inferences that can be applied when presented with novel data [1]. Generative models function by similarly analysing datasets, but with a view to produce new data resembling the real original data. In healthcare, deductive machine learning models have captured academic, clinical and media attention due to their increasing ability to gain population health insights from large datasets and use these to inform health policy. Generative artificial intelligence (AI) is a much newer phenomenon and the ability to create synthetic data has use cases including dataset augmentation and data privacy [2–4]. Generative AI models, including generative adversarial networks, function by creating synthetic datasets composed of uniquely generated ‘fake’ datapoints, which at an aggregate level maintain all the patterns of the original dataset. It is already being considered that AI-generated synthetic datasets may begin to be used in place of real datasets to train deductive machine learning models [5]. The main advantages of doing so are that anonymous synthetic data can be shared instead of real data and the size of the datasets can be artificially increased. These advantages rest upon the assumption that the synthetic data is representative of the original dataset and patterns are preserved. Several methods of assessing the fidelity of synthetic data have been proposed, including simplistic Turing tests, histogram analysis and comparing the outputs of research analysis with those from real data [6, 7]. The lack of uniformly accepted standards for assessing synthetic data is a major limitation to the field [8]. This is further complicated by the fact that data must maintain high fidelity with the original data, but must be sufficiently different that the datapoints are genuinely non-identifiable.

This study presents a series of machine learning models trained to predict blood pressure of individuals within the National Diet and Nutrition Study (NDNS), based on simple variables [9]. The prediction of blood pressure using machine learning is an area of research which has gained attention recent years, with models usually being trained on electrocardiogram (ECG) or photoplethysmography (PPG) data, though there are examples of models trained on risk factors [10, 11]. The results of machine learning models are compared with those produced by machine learning models trained on synthetic datasets, of varying sizes, representative of the National Diet and Nutrition Study. In each case, the data is tested on the same sample of real data, but is trained on either real or synthetic datapoints.

## Methods

### Study design and dataset

This was a cross-sectional retrospective machine-learning study. This study uses data from the National Diet and Nutrition Study Rolling Programme (NDNS) (2008–2019). Ethical approval for the NDNS was obtained from the Oxfordshire A Research Ethics Committee and the Cambridge South NRES Committee (Ref. No. 13/EE/0016). In this analysis, we use data on adults aged from 18 to 70 years, combined from the first eight years (2008–2019) of the NDNS to provide a sufficiently large sample size for analysis (Table 1). An upper age limit of 70 years was applied due to the likelihood of comorbidities affecting blood pressure in the elderly population. Participants who reported taking anti-hypertensive medications were also excluded from the analysis. Mean arterial pressure, which is the average arterial pressure throughout one cardiac cycle, was calculated as the outcome variable, using the following equation [12]:

$$Average\;blood\;pressure=diastolic\;blood\;pressure+0.3333333\;x\left(systolic-diastolic\;blood\;pressure\right)$$

**Table 1:** Descriptive statistics of the study population, grouped by age. Only age, sex, height and weight were ultimately used in the machine learning models as predictive variables. All variables were, however, used for imputing missing data and constructing synthetic datasets.

		Age category			
		18 to 35 (n = 933)	36 to 49 (n = 941)	50 to 70 (n = 958)	Total (n = 2832)
**Sex**	**Male**	377	366	396	1139
	**Female**	556	575	562	1693
**Ethnicity**	**White**	839	863	923	2625
	**Mixed ethnic group**	20	8	7	35
	**Black or Black British**	23	24	10	57
	**Asian or Asian British**	38	31	11	80
	**Any other group**	13	15	7	35
**Smoking status**	**Never a regular smoker**	587	563	534	1684
	**Ex-smoker**	136	192	296	624
	**Current smoker**	210	186	128	524
**Marital status**	**Not married**	644	235	110	989
	**Married**	247	541	572	1360
	**Civil partnership**	42	165	276	483
**Socioeconomic status**	**Managerial and professional occupations**	367	484	416	1267
	**Intermediate occupations**	200	170	224	594
	**Routine and manual occupations**	343	272	308	923
	**Never worked and other**	23	15	10	48
**Total screen time (sd) (hours)**		76.4 (19.1)	70.8 (17.1)	71.5 (17.0)	72.9 (17.9)
**Frequency of takeaway meals (sd)**		3.9 (0.8)	4.0 (0.8)	4.4 (0.7)	4.1 (0.8)
**Average sleep duration (sd) (hours)**		7.6 (1.1)	7.2 (1.1)	7.0 (1.2)	7.2 (1.2)
**Height (sd) (cm)**		169.7 (9.4)	168.0 (9.2)	166.9 (9.1)	168.2 (9.3)
**Weight (sd) (kg)**		74.4 (17.2)	78.4 (16.3)	78.0 (15.8)	76.9 (16.5)
**Average blood pressure (sd) (mmHg)**		85.3 (9.7)	90.2 (11.5)	94.0 (11.8)	89.9 (11.6)

There were 55 datapoints missing socioeconomic status, 3 datapoints missing smoking status, 707 datapoints missing screen time, 1 datapoint missing takeaway meal frequency, 1 datapoint missing ethnicity, 96 datapoints missing sleep duration, 56 datapoints missing height and 71 datapoints missing weight. Missing datapoints were imputed by the multiple imputation package ‘missForest’ with all variables being used as predictors [13].

### Statistical analysis and machine learning

The NDNS data was randomly split into a training dataset (85%, n = 2408) and a testing dataset (15%, n = 424). The testing dataset was reserved entirely for testing machine learning model predictive performance. A selection of variables from the NDNS were isolated and directed acyclic graphs were drafted to consider whether a causal relationship with blood pressure may be plausible. These variables included: age, sex, ethnicity, marital status, smoking status, socioeconomic status, total weekly screen time, how often takeaway meals are consumed weekly, average nightly sleep duration, height and weight. Average weekly sleep duration was calculated based on the amount of self-reported sleep over the past seven days, in a method described previously [14]. These eleven variables, in the training dataset, were fed into a recursive feature elimination model by tenfold cross-validation to identify the optimal combination of variables for the predictive models, based on minimisation of mean absolute error. The combination of variables which were identified to produce the optimum results from the training data with the fewest number of variables were: age, weight, height and sex. Dummy variables were constructed by one-hot encoding for each value of the categorical variables, creating a total number of five variables. All data, apart from the blood pressure outcome, were scaled to be on the interval between zero and one to prevent disproportionate importance being assigned to variables with larger ranges of values. The scaling transform learnt on the training data was applied to the test data. All analyses were performed using R (version 4.2.2) [15]. Tenfold cross-validation was used to train models, with mean absolute error (MAE) used as the optimisation metric. Three models were constructed using the caret package in R: Bayesian generalised linear regression (Caret: bayesglm), random forest (Caret: rf) and neural network (Caret: nn). The models were chosen to incorporate a range of high-performing regression predictive models that can easily be reproduced using open-source software packages. The residuals of each model for each dataset were compared using the Wilcoxon signed rank test value with continuity correction, at a significance level of p = 0.05.

### Synthetic data

**Fig 1:**
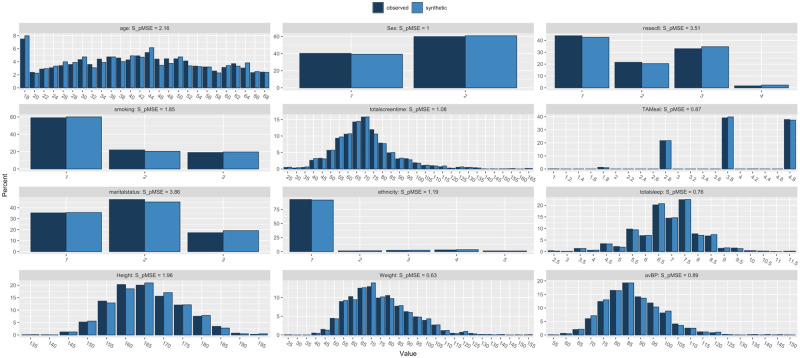
Histogram comparison for each variable comparing the aggregate demographic characteristics of the real training dataset (n = 2408) against synthetic dataset A (n = 2408).

**Fig 2:**
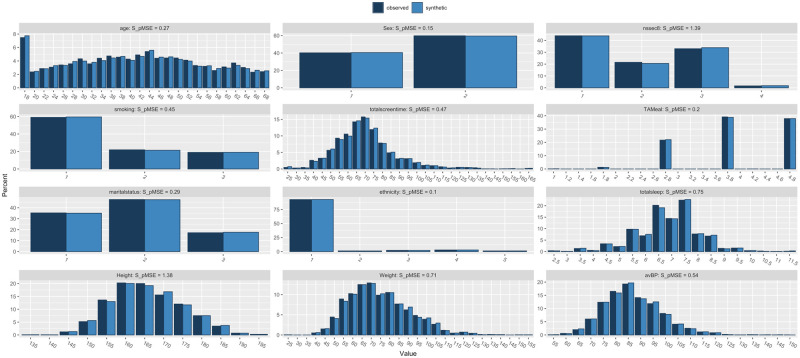
Histogram comparison for each variable comparing the aggregate demographic characteristics of the real training dataset (n = 2408) against synthetic dataset B (n = 4816).

Three synthetic datasets were constructed, each based on the original training dataset. No data from the test dataset was leaked into the generation of the synthetic datasets. The *synthpop* library in R was used to produce two datasets of sizes: n = 2408 and n = 4816 [16]. A third dataset (n = 4816) was also used in the analysis, which consisted of the real data training set and the synthetic dataset (n = 2408) combined. Fig 1 illustrates the basic demographic analyses of synthetic dataset A (n = 2408) datasets compared to the real training dataset in histogram form. Fig 2 illustrates the comparison between synthetic dataset B (n = 4816) and the real training dataset, with both synthetic datasets demonstrating high fidelity at an aggregate level. The same machine learning analysis described above was applied to each of the synthetic datasets with the same test dataset used for the testing of all models. Identical pre-processing was also applied to the synthetic datasets and the same variables were isolated for analysis.

## Results

Of the 15655 participants who took part in years 1–11 of the NDNS, 7697 had blood pressure values recorded. Of these, 3256 were aged between 18 and 70 years old. A further 410 participants were excluded for taking blood pressure lowering medication, leaving a sample size of 2832 for the study (Table 1).

Two synthetic datasets were constructed, of different sizes. Table A was the same size as the training dataset comprised of real data (n = 2408), Table B was double the size (n = 4816). The datasets were generally of high fidelity, when compared to the real training dataset with the aggregate descriptive analysis comparing each variable displayed in Table 2.

**Table 2:** Statistical analysis comparing synthetic data tables to the real training dataset (n = 2408). Presented are propensity score mean-squared-error and standardised ration of propensity score mean-squared error.

	Synthetic data table A		Synthetic data table B	
	n = 2408		n = 4816	
	pMSE	S_pMSE	pMSE	S_pMSE
**Age**	0.000225	2.16	0.000011	0.27
**Sex**	0.000026	1.00	0.000001	0.15
**Socioeconomic status**	0.000273	3.51	0.000043	1.39
**Smoking status**	0.000096	1.85	0.000009	0.45
**Total weekly screen time**	0.000112	1.08	0.000019	0.47
**Frequency of eating takeaway meals**	0.000045	0.87	0.000004	0.20
**Marital status**	0.0002	3.86	0.000006	0.29
**Ethnicity**	0.000124	1.19	0.000004	0.10
**Total weekly sleep duration**	0.000079	0.76	0.000031	0.75
**Height**	0.000204	1.96	0.000057	1.38
**Weight**	0.000065	0.63	0.000029	0.71
**Average blood pressure**	0.000093	0.89	0.000022	0.54

The performances of the machine learning models are shown in Table 3. The three model types were comparable in their results. Wilcoxon signed rank test with continuity correction to compare the residuals of each model. It can be seen that for each model type, algorithms trained comparably, regardless of whether they were trained on real data, synthetic data or the augmented real dataset.

**Table 3:** Results of the machine learning models, trained on real or synthetic datasets. Each was tested on the same test dataset (real data). None of the p-values were <0.05.

Machine learning model	RMSE	Rsquared	MAE	Residuals	Wilcoxon signed rank test value (V)	p-value
**Bayes linear regression model (real data)**	10.47	0.22	8.28	46483	**-**	
**Bayes linear regression model (synthetic data table A)**	10.46	0.22	8.26	46361	47374	0.36
**Bayes linear regression model (synthetic data table B)**	10.45	0.22	8.25	46346	45981	0.71
**Bayes linear regression model (real data and synthetic data table A combined)**	10.46	0.22	8.27	46352	48244	0.21
**Neural network model (real data)**	10.35	0.24	8.19	45425	**-**	
**Neural network model (synthetic data table A)**	10.36	0.24	8.16	45466	44606	0.86
**Neural network model (synthetic data table B)**	10.33	0.24	8.14	45259	45261	0.93
**Neural network model (real data and synthetic data table A combined)**	10.31	0.25	8.12	45077	45761	0.78
**Random forest model (real data)**	10.55	0.21	8.33	47214	**-**	
**Random forest model (synthetic data table A)**	10.52	0.21	8.20	46920	46123	0.67
**Random forest model (synthetic data table B)**	10.71	0.18	8.30	48619	45214	0.95
**Random forest model (real data and synthetic data table A combined)**	10.49	0.22	8.22	46620	49952	0.05

## Discussion

Algorithms trained on synthetic data performed comparably to those trained on real data with no significant differences found between the two types. Furthermore, when comparing the descriptive statistics of the real and synthetic datasets, there was minimal difference between them at an aggregate level. Together, these findings support the hypothesis that synthetic data can be used to train machine learning algorithms with the intention that they be tested on real data. All algorithms were able to predict blood pressure with a mean absolute error of approximately 8.2mmHg. The only variables used to achieve this were: age, sex, weight and height. This feat was achieved based on the use of recursive feature elimination to ensure that unnecessary variables were not being including in the model. In predicting blood pressure, this study represents a minimalistic approach to variables used in training the machine learning models, which increases its applicability in the real world.

There have been previous research efforts to predict blood pressure using other elements of a patient’s healthcare record. This has often involved electrocardiogram (ECG) analysis and photoplethysmography (PPG) [17, 18]. Whilst capable of performing impressively, for example Wang et al achieved an MAE of 4.02 for systolic blood pressure, these are less applicable at a population level due to the need for physical devices and monitoring. Another study conducted by Nath et al produced an MAE of approximately 10mmHg using age, dual‐energy X‐ray absorptiometry (DXA) measured body composition parameters, BMI, and waist circumference [19]. Other studies have sought to simply predict whether an individual has hypertension or not, using similar methodologies. In these cases, accuracies of predictions have roughly been in the range of 80–90% for high performing models [20, 21]. Studies using population level variables such as in this study have typically focussed on predicting the presence or absence of hypertension. Indeed, this study represents one of the first studies, if not the first study to predict the actual blood pressure value using descriptive clinical data without the need for EEG or PPG monitoring [10]. Even of those studies which have used descriptive clinical data to predict the presence of hypertension, typically these have used specific variables relating to blood pressure, including doctor’s perception of their blood pressure and whether they have measured their blood pressure [21]. Predicting blood pressure is a potentially important research area as it could give rise to targeted public health measures to optimise one of the most well recognised predictors of cardiovascular disease and all-cause mortality [22, 23]. As well as directing influencing the management of hypertension at a population level, this realm of research has the potential to improve our understanding of the disease process underlying hypertension as we begin to understand which variables can be predictive of high blood pressure. By combining multimodal data, for example clinical, genetic and physiological datapoints we may be able to yield more accurate predictions in the future [24]. The ability of synthetic data to inflate sample sizes in training data is an area for future research. In this study, there was no significant difference between the performance of models trained on smaller (n = 2408) or larger (n = 4816) datasets. This suggests that the real data sample size of 2408 was sufficient to train the algorithms. Future research could attempt to train algorithms on smaller sample sizes and assess, in granular detail, the relationship of synthetic data sample size with model accuracy.

Research into synthetic data is still in its infancy, with studies beginning to emerge suggesting the potential of synthetic data to train machine learning models to produce comparable results to the training on real data. Although this study used data that was already publicly available, it has been suggested that the same method of synthetic data generation used here could be used to help researchers release open-access datasets in a synthetic version of the actual confidential data used for studies [25]. Data confidentiality and ownership is an important ethical barrier to the implementation of artificial intelligence in healthcare, with synthetic data emerging as a potential solution [26]. A practical example if the use of synthetic data to replicate the results of a published stage III colon cancer trial secondary analysis, with high concordance between the results of models based on real and synthetic data [27]. Generative forms of artificial intelligence have also been used to create other forms of synthetic data, for example time-series data within electroencephalogram (EEG) signals and to selectively generate fundus photos from underrepresented groups to re-balance retinal imaging data [28, 29]. However, despite the well-recognised advantages of synthetic data, concerns are beginning to materialise. This includes the risk of data being used maliciously, or as a means of bypassing data protection legislation [30]. More broadly, there are ethical implications involved with this line of research. For example, if insurance providers are able to predict a patient’s blood pressure or other health characteristics, they could use this information to adjust premiums.

### Limitations

The focus of this study was to compare the performance of machine learning models based on the type of data they were trained on. The study used data that is not representative of the United Kingdom general population. Although the NDNS does include survey weights which, if used, would enable analysis representative of the national population these were not used due to incompatibility with the analysis chosen and the amount of data removed due to ineligibility for the analysis. Further research would be required to validate the use of machine learning algorithms to predict blood pressure from the variables presented in this study. Though this research indicated this may be possible, it did not test, nor present evidence of, generalisability when applied to a larger population. Therefore, this paper does not draw substantive conclusions about the use of these variables to predict blood pressure. Our conclusions instead focus upon the use of synthetic data to produce results comparable to those generated using real data. This study did not explore the reidentification risk of using synthetic data. This is a concern with the use of synthetic data to replace real datasets. Whilst it is important to ensure that synthetic datasets maintain a high degree of fidelity with the original data and analyses can be performed comparably, there is also a risk that the synthetic datasets may be so similar that the original datapoints can be identified.

### Concluding remarks

The purpose of this study was to explore the comparability of machine learning algorithms trained on real and synthetic data to predict blood pressure using population level clinical data. All algorithms performed comparably to previous research efforts aimed at predicting blood pressure. In order for models trained on synthetic data to perform comparably to those from real data, they required a larger dataset. Generative AI is able to produce datasets of theoretically unlimited sizes and this study suggests that there may be a role to use synthetic data in place of real data when training machine learning algorithms on population health datasets.
